# Spatial Variation of Soil Respiration in a Cropland under Winter Wheat and Summer Maize Rotation in the North China Plain

**DOI:** 10.1371/journal.pone.0168249

**Published:** 2016-12-15

**Authors:** Ni Huang, Li Wang, Yongsen Hu, Haifeng Tian, Zheng Niu

**Affiliations:** 1 The State Key Laboratory of Remote Sensing Science, Institute of Remote Sensing and Digital Earth, Chinese Academy of Sciences, Beijing, China; 2 University of Chinese Academy of Sciences, Beijing, China; Pacific Northwest National Laboratory, UNITED STATES

## Abstract

Spatial variation of soil respiration (*R*_*s*_) in cropland ecosystems must be assessed to evaluate the global terrestrial carbon budget. This study aims to explore the spatial characteristics and controlling factors of *R*_*s*_ in a cropland under winter wheat and summer maize rotation in the North China Plain. We collected *R*_*s*_ data from 23 sample plots in the cropland. At the late jointing stage, the daily mean *R*_*s*_ of summer maize (4.74 μmol CO_2_ m^-2^ s^-1^) was significantly higher than that of winter wheat (3.77μmol CO_2_ m^-2^ s^-1^). However, the spatial variation of *R*_*s*_ in summer maize (coefficient of variation, CV = 12.2%) was lower than that in winter wheat (CV = 18.5%). A similar trend in CV was also observed for environmental factors but not for biotic factors, such as leaf area index, aboveground biomass, and canopy chlorophyll content. Pearson’s correlation analyses based on the sampling data revealed that the spatial variation of *R*_*s*_ was poorly explained by the spatial variations of biotic factors, environmental factors, or soil properties alone for winter wheat and summer maize. The similarly non-significant relationship was observed between *R*_*s*_ and the enhanced vegetation index (EVI), which was used as surrogate for plant photosynthesis. EVI was better correlated with field-measured leaf area index than the normalized difference vegetation index and red edge chlorophyll index. All the data from the 23 sample plots were categorized into three clusters based on the cluster analysis of soil carbon/nitrogen and soil organic carbon content. An apparent improvement was observed in the relationship between *R*_*s*_ and EVI in each cluster for both winter wheat and summer maize. The spatial variation of *R*_*s*_ in the cropland under winter wheat and summer maize rotation could be attributed to the differences in spatial variations of soil properties and biotic factors. The results indicate that applying cluster analysis to minimize differences in soil properties among different clusters can improve the role of remote sensing data as a proxy of plant photosynthesis in semi-empirical *R*_*s*_ models and benefit the acquisition of *R*_*s*_ in cropland ecosystems at large scales.

## Introduction

Soil respiration (*R*_*s*_) is an important process in the carbon flux between the terrestrial ecosystem and the atmosphere, and plays a critical role in global carbon cycling [1], [2]. *R*_*s*_ can be divided into autotrophic and heterotrophic respiration based on different biological sources [3]. Autotrophic respiration, also known as root respiration, mainly depends on the supply of photosynthetic substrates [4], [5], [6]. Heterotrophic respiration is the sum of microbial decomposition of soil organic matter [7]. Generally, autotrophic and heterotrophic respirations fluxes are regulated by different mechanisms and interact over different temporal and spatial scales, resulting in the accurate prediction of *R*_*s*_ difficult [8], [9]. Factors affecting *R*_*s*_ must be elucidated to improve the current carbon cycle models and estimate carbon efflux from ecosystems to the atmosphere [10], [11].

Environmental factors, such as soil temperature and soil moisture, are important abiotic regulators of *R*_*s*_ [12], [13], [14], [15]. Plant productivity or photosynthesis capacity proxies, such as leaf area index (LAI), canopy chlorophyll content (Chl_canopy_), and biomass, can become the dominant biotic regulators of *R*_*s*_ [16], [17], [18]. This phenomenon occurs especially when *R*_*s*_ is obtained during the time of high root respiration [19], [20] and high rhizodeposition [21], [22], such as the peak growing season of vegetation [23].

Compared with studies on temporal variation in *R*_*s*_, relatively few studies have explored the spatial variation of *R*_*s*_. A few reports have contended that spatial patterns of *R*_*s*_ may be controlled more by photosynthesis and productivity than by soil temperature [4], [24], [25], [26]. Temporal patterns of *R*_*s*_ have been simulated using continuous records of temperature, moisture, and other variables [12], [27], [28], [29]. Compared with methods for estimating the temporal variation of *R*_*s*_, methods for quantifying spatial variation of *R*_*s*_ are limited and difficult [30]. The spatial difference in *R*_*s*_ within a site and between sites is often not explained by climatic variables; instead, the difference is modulated by gradients in the biological activity and differences in the soil properties [26], [27], [31], [32]. These features may provide a basis to design field experiments and conduct data analysis to improve the estimation of soil CO_2_ emission from an ecosystem.

Researchers have used various statistical methods to disentangle the cross-correlated controlling factors of *R*_*s*_ from one another. For example, a structural equation modeling approach was used to identify direct and indirect affecting factors of *R*_*s*_ in alpine meadow [17] and maize fields [33]. Cluster analysis was performed to identify possible groups of sites where soil CO_2_ concentration could be affected by different factors [34]. Compared with the structural equation modeling approach, cluster analysis is simple because it does not require significant correlation among the analyzed variables and does not depend on the subjective experience and prior knowledge of the analyst [35], [36], [37].

Remote sensing technology is gradually gaining importance in research on the global carbon cycle because of its spatially extensive coverage and low cost [38], [39], [40]. However, the application of remote sensing data in studies of *R*_*s*_ is not always practical and presents several uncertainties. Our previous studies examined the possibility of using remotely sensed data to estimate *R*_*s*_ in croplands [18], grasslands [23], and forests [33]. These studies established the feasibility of remotely-sensed spectral vegetation indices (VIs) in *R*_*s*_ analysis. VIs representing vegetation greenness were correlated with proxies of plant productivity such as gross primary production and leaf area index. However, covariation of plant productivity and other factors (i.e., soil temperature, moisture, and soil properties) [41], [42] complicates the explanation of the relationships between VIs and *R*_*s*_. When the spatial variations of environmental factors (i.e., temperature and soil moisture) at a county scale were negligible, a model incorporating VIs and soil organic carbon (SOC) content produced satisfactory accuracy for predicting *R*_*s*_ during the peak growing season of maize [43]. Hence, the relationships between VIs and *R*_*s*_ were affected by soil properties. To further improve the role of remotely sensed VIs in *R*_*s*_ estimation at the spatial scale, scholars must determine the mechanism through which soil properties regulate the relationships between VIs and *R*_*s*_. The present study employs cluster analysis to analyze the spatial variation of *R*_*s*_ in a cropland under winter wheat and summer maize rotation, examines the relationship between *R*_*s*_ and spectral vegetation index in each cluster, and investigates how the soil properties regulate this relationship.

## Materials and methods

### Ethics statement

**Fig 1:**
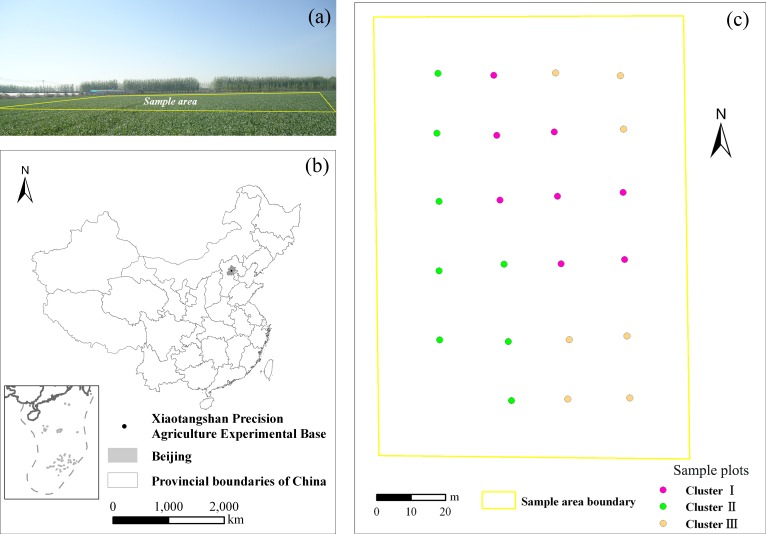
Location of study site and spatial distributions of sample plots. The figure in the left bottom corner of Fig 1 is similar to Figure 1 in the reference [43] but not identical to the Figure 1 in the reference [43]. The box in the left bottom corner refers to the South China Sea islands.

No specific permissions were required for the 23 sample plots in this study. We confirmed that the field studies did not involve endangered or protected species. The specific location of the sample plots is provided in the manuscript (Fig 1).

### Site description

The field experiment was conducted at Xiaotangshan Precision Agriculture Experimental Base, Changping District, Beijing, North China (40° 10.6′ N, 116° 26.3′ E). This experimental base has been operational since 2001 and is used for precision agriculture research. This site is located in a warm temperate zone with a mean annual rainfall of 507.7 mm and a mean annual temperature of 13°C [44]. The soil at this site is a silt-clay loam [44]. The double cropping system of winter wheat (*Triticumaestivum* L.) and summer maize (*Zeamays* L.) is the farming practice at this site and is the dominant farming style in the North China Plain. Winter wheat is usually sowed in October and harvested in June of the following year. Approximately 10 cm-high wheat residuals are left on the field surface after harvest. Summer maize is sowed in June without tillage and then harvested in October. When precipitation is scarce, crops are irrigated depending on soil water status.

### Experimental design

A very flat sample area was established at this site with slopes less than 1°and a size of 140 m × 100 m. Land leveling was conducted in this sample area about 2 years ago (November 2013). A single crop cultivar was then planted in this area, which is under uniform water and fertilizer management. Before land leveling, the sample area was subjected to different treatments, such as using different fertilizers and crop cultivars to meet different research needs [44], [45], [46]. Therefore, soil properties showed spatial variations in the sample area because of long-term differences in farm managements (i.e., fertilizer, irrigation and cultivar), which might have led to variations in the vegetation growth and *R*_*s*_. To determine the spatial variation of *R*_*s*_, we employed a grid sampling method, where the distance between each sample plot was approximately 20 m (Fig 1). This design was in accordance with the research results on spatial autocorrelation of soil properties at this site [45].

The field experiments were conducted at two continuous sunny days at the late jointing stages of winter wheat (April 20 to 22, 2015) and summer maize (August 3 to 5, 2015), which mostly corresponded to the period of the highest biological activity because of the maximum crop growth rate [18]. On April 13, 2015, the sample plots were fully irrigated to meet the water requirements of winter wheat growth. A heavy rain event occurred on July 27, 2015, approximately 1 week before the summer maize experiment. Therefore, the soil water content was considered to be suitable for crop growth at the time when we conducted the two filed experiments.

Field experiments were conducted at 23 sample plots (Fig 1). We conducted the summer maize experiment at the same sample plots where the winter wheat experiment was conducted using high-precision GPS positioning. Each plot size was 1.5 m × 1.5 m. In each plot, we measured the variables that might explain the spatial variation of *R*_*s*_: these variables include (1) *R*_*s*_; (2) biotic factors measured by aboveground biomass (AGB), leaf area index (LAI), and canopy chlorophyll content (Chl_canopy_); (3) environmental factors encompassing soil water content at 0–20 cm depth (SWC_20_) and soil temperature at 10 cm depth (T_s10_); (4) soil property factors, including soil total nitrogen (STN) content, soil total carbon (STC) content, soil carbon/nitrogen (C/N), and soil organic carbon (SOC) content; and (5) canopy spectral reflectance of winter wheat and summer maize. It is noteworthy that we only measured soil property factors during the winter wheat experiment because of the short interval between the winter wheat and summer maize experiment (3 months). We assumed that the soil properties in such a short time could be considered constant. Therefore, the measurement data for soil properties were used to analyze the spatial variation of *R*_*s*_ in the winter wheat and summer maize.

### Measurements of soil respiration and environmental factors

In each sample plot, *R*_*s*_ was measured using a *R*_*s*_ chamber (6400–09; LiCor, Lincoln, Nebraska, USA) connected to a portable photosynthesis system (LI-6400; LiCor, Lincoln, Nebraska, USA). The *R*_*s*_ chamber was mounted on a PVC soil collar that was sharpened at the bottom. Four soil collars were randomly distributed in each plot for the winter wheat experiment. Six soil collars were installed in each plot for the summer maize experiment. Each *R*_*s*_ measurement was performed between 09:00 h and 12:00 h (local time) because fluxes measured during this time interval usually represent the daily mean flux [18]. *R*_*s*_ measurement procedures, soil collar placement, and *R*_*s*_ data processing were described in previous studies [18], [43]. After the *R*_*s*_ measurement on the PVC soil collar in each plot (S1 Table), the soil temperature at 10 cm depth (T_s10_) and soil moisture at 0–20 cm (SM_20_) were measured in the collar to minimize sample difference. Detailed procedures for soil temperature and soil moisture measurements were previously described by Huang *et al*. [43].

### Canopy reflectance measurements and vegetation index calculation

Canopy reflectance was measured after the installation of soil collars. A portable spectroradiometer (FS-FR2500, ASD, USA) was used to measure winter wheat and summer maize canopy radiance between 350 and 2500 nm with a 1 nm waveband width. The procedures for canopy reflectance measurements were described in detail by Huang *et al*. [18]. Based on the measured canopy reflectance data, three VIs, namely, normalized difference vegetation index (NDVI), red edge chlorophyll index (CI_red edge_), and enhanced vegetation index (EVI), were calculated to analyze their relationships to the biotic factors of winter wheat and summer maize. Three formulas used for the calculation of these VIs were described by Huang *et al*. [18].

### Biotic factor measurements

LAI was measured with a LAI-2000 plant canopy analyzer (LI-COR Inc., Lincoln, Nebraska, USA). In each plot, five representative positions were selected for LAI measurement, and two repeated measurements were performed at each position. Chl_leaf_ was obtained with a portable chlorophyll meter (SPAD-502, New Jersey, USA). The Chl_leaf_ measurement procedures and Chl_canopy_ calculation were described in detail by Huang *et al*. [18]. AGB was measured by randomly harvesting the aboveground fresh winter wheat plants in three subplots (0.2 m×0.2 m) and three maize plants in each plot. The fresh samples were oven dried at 65°C until the mass of the sample became constant. AGB measurement damaged the samples. Thus, we conducted this measurement when all the other measurements were finished. To reduce spatial sampling and measurement errors, we averaged the LAI, Chl_leaf_, and AGB derived from each plot for both winter wheat and summer maize for further analysis.

### Soil property measurements

Soil inside the four PVC collars in each plot was destructively sampled after measuring *R*_*s*_, soil temperature, and soil moisture in the winter wheat experiment. The collected soil samples were stored at room temperature and rapidly transported to the nearby laboratory (approximately 200 meters from the sampling site) for analysis. Soil sampling procedures and soil sample processing were described elsewhere [43]. SOC content was estimated by the standard Mebius method [47]. STN and STC content were measured by an elemental analyzer (Isoprime-EuroEA3000, Milan Italy). Soil C/N was calculated from the ratio of the STC and STN content.

### Data analysis

Correlation analysis was employed to examine the relationships among *R*_*s*_, biotic factors, environmental factors, and soil properties. The coefficient of variation (CV) was used to represent the spatial variation of *R*_*s*_ and its various affecting factors. The relationships between biotic factors (i.e. LAI, AGB, and Chl_canopy_) and VIs (i.e., NDVI, CI_red edge_, and EVI) were examined using regression analysis. The optimum VI was selected based on the determination coefficient (R^2^).

Previous studies [45], [46] revealed that soil properties in our experimental area exhibited spatial variance. Spatial clustering of sample plots based on soil property factors is advisable to detect the possible confounding effects of soil properties on the relationship between *R*_*s*_ and other biotic or abiotic factors, and elucidate the relationship between *R*_*s*_ and VIs. In the present study, cluster analysis was performed based on the soil property factors to quantify the similarity in the 23 sample plots. Hanesch *et al*. [48] demonstrated that using all the variables causes over-information in cluster analysis and leads to insufficiently distinguishable samples from one another. The high correlation among input variables will over-represent one variable and bias the cluster results [49], [50]. Correlation analysis of the soil properties (Table 1) demonstrated that the SOC content highly correlated with the STN content (Pearson’s correlation coefficient r = 0.83, p<0.001) and STC content (r = 0.86, p < 0.001). Soil C/N displayed no significant correlation (p > 0.05) with the SOC, STN, and STC content (Table 1). Therefore, the SOC content and soil C/N were considered in the cluster analysis. Moitinho *et al*. [51] also demonstrated that SOC and soil C/N ratio are the two most important soil property variables that affect spatial variation of *R*_*s*_ in a sugarcane field. Before cluster analysis, the variables were standardized using the methods of Jiang *et al*. [37]. Based on the results of the cluster analysis, linear regression between *R*_*s*_ and optimal VI was used to detect the possible relationship between *R*_*s*_ and the photosynthesis proxy factor derived from remote sensing data in each cluster. One-way ANOVA with the least significant difference (LSD) multiple comparison test was used to analyze differences in *R*_*s*_, biotic factors, environmental factors, and soil properties among different clusters. All the statistical analyses were performed with the Statistical Package for the Social Sciences (SPSS, Chicago, Illinois, USA).

**Table 1:** Correlation coefficients among soil respiration (*R*_*s*_), leaf area index (LAI), aboveground biomass (AGB), canopy chlorophyll content (Chl_canopy_), soil water content at 0–20 cm depth (SWC_20_), soil temperature at 10 cm depth (T_s10_, °C), soil total nitrogen (STN) content, soil total carbon (STC) content, soil carbon/nitrogen (C/N) ratio, and soil organic carbon (SOC) content at the late jointing stage of winter wheat and summer maize in North China plain.

	*R*_*s*_	LAI	AGB	Chl_canopy_	SWC_20_	T_s10_	STN content	STC content	Soil C/N	SOC content
*R*_*s*_	1	**0.25**	**0.42**	**0.24**	**0.35**	**0.3**	**-0.3**	**-0.35**	**0.02**	**-0.49**
LAI	0.21	1	0.92***	0.99***	**-0.06**	**-0.01**	**0.03**	**0.21**	**0.26**	**0.17**
AGB	0.33	0.88***	1	0.92***	**-0.06**	**0.05**	**-0.15**	**0.03**	**0.3**	**-0.03**
Chl_canopy_	0.30	0.82***	0.78***	1	**-0.1**	**0.02**	**0.05**	**0.23**	**0.26**	**0.19**
SWC_20_	-0.34	0.10	-0.10	0.06	1	**-0.06**	**-0.11**	**-0.43**	**-0.47**	**-0.34**
T_s10_	0.28	-0.26	-0.11	-0.16	-0.51	1	**-0.06**	**-0.08**	**-0.01**	**-0.01**
STN content	0.03	0.21	0.16	0.47	-0.29	0.15	1	0.78***	**-0.41**	0.83***
STC content	0.02	0.40	0.28	0.45	-0.30	-0.11	0.78***	1	**0.24**	0.86***
Soil C/N	-0.01	0.30	0.19	-0.02	0.07	-0.42	-0.41	0.24	1	**-0.05**
SOC content	0.23	0.29	0.28	0.43	-0.54	0.29	0.83***	0.86***	-0.05	1

Significance levels

***p < 0.001

Bold signal means the correlation analysis results for winter wheat, and the no mark values describe the results for summer maize.

## Results

### Spatial variations of soil respiration, biotic, and abiotic factors

Based on the spatially measured data from 23 sample plots in the winter wheat experiment, the daily mean *R*_*s*_ at the late jointing stage of winter wheat was 3.77 μmol CO_2_ m^-2^ s^-1^ with a range of 2.40 μmol CO_2_ m^-2^ s^-1^ to 4.88 μmol CO_2_ m^-2^ s^-1^. The CV of *R*_*s*_ for winter wheat was 18.5% (Table 2). Biotic factors, such as LAI, AGB, and Chl_canopy_, displayed high spatial variability with CV ranging from 18.2% to 25.1%. Compared with the soil temperature (T_s10_, CV = 4.8%), the soil water content (SWC_20_, CV = 15.7%) showed larger spatial variation. Among soil property factors, SOC content demonstrated higher spatial variation (15.1%) than the other three soil properties (7.2%–10.8%; Table 2).

**Table 2:** Descriptive statistics for soil respiration, biotic and abiotic factors.

	Winter wheat					Summer maize				
	Min	Max	Mean	SD	CV(%)	Min	Max	Mean	SD	CV(%)
***R***_***s***_	2.40	4.88	3.77	0.71	18.5	3.74	5.70	4.74	0.58	12.2
***Biotic factors***										
LAI	1.61	4.54	3.03	0.76	25.1	2.02	3.48	2.89	0.42	14.5
AGB	0.25	0.54	0.42	0.08	18.2	0.11	0.35	0.26	0.06	24.5
Chl_canopy_	0.72	2.01	1.32	0.33	25.0	0.58	1.96	1.16	0.28	24.1
***Environmental factors***										
SWC_20_	22.2	38.4	26.6	4.2	15.7	33.5	42.7	36.8	5.0	10.8
T_s10_	12.7	14.9	13.6	0.7	4.8	22.1	25.2	23.5	0.8	3.5
***Soil property factors***										
STN content	0.12	0.21	0.17	0.02	10.8					
STC content	0.87	1.28	1.12	0.11	10.1					
Soil C/N	5.94	7.67	6.77	0.49	7.2					
SOC content	0.87	1.25	1.09	0.16	15.1					

*R*_*s*_ is the soil respiration (μmol CO_2_ m^-2^ s^-1^), LAI is the leaf area index, AGB is the aboveground biomass (kg m^-2^), Chl_canopy_ is the canopy chlorophyll content (g m^-2^), SWC_20_ is the soil water content at 0–20 cm depth (%), T_s10_ is the soil temperature at 10 cm depth (°C), STN content is the soil total nitrogen content (%), STC content is the soil total carbon content (%), soil C/N is the soil carbon/nitrogen ratio, and SOC content is the soil organic carbon content (%). SD is the standard deviation; CV is the coefficient of variation.

At the late jointing stage of summer maize, the daily mean *R*_*s*_ was 4.74 μmol CO_2_ m^-2^ s^-1^ with a minimum of 3.32 μmol CO_2_ m^-2^ s^-1^and a maximum of 5.70 μmol CO_2_ m^-2^ s^-1^. The daily mean *R*_*s*_ was significantly higher than the corresponding values for winter wheat (p < 0.05). The spatial variation of *R*_*s*_ showed difference in the same field when winter wheat and summer maize were planted. The CV of *R*_*s*_ for summer maize (12.2%) was lower than that for winter wheat (18.5%). The similar trend in CV was observed for environmental factors (SWC_20_ and T_s10_). The CVs of LAI and Chl_canopy_ for summer maize were consistently lower than their corresponding values for winter wheat.

### Relationships between soil respiration and biotic or abiotic factors

During the late jointing stage of winter wheat and summer maize, none of the factors were statistically significant important in explaining the spatial variation of *R*_*s*_ based on the Pearson’s correlations between *R*_*s*_ and various biotic or abiotic factors in the 23 sample plots (Table 1). Therefore, the spatial variation of *R*_*s*_ was poorly explained by the spatial variations of biotic factors, environmental factors, or soil properties alone for winter wheat and summer maize. The STN, STC, and SOC content had relatively high correlation coefficients, which ranged from 0.78 to 0.86. The correlations between soil C/N and other soil properties were not statistically significant (p > 0.05). Significant correlations were found among LAI, AGB, and Chl_canopy_ for winter wheat and summer maize.

### Cluster analysis of soil properties

**Fig 2:**
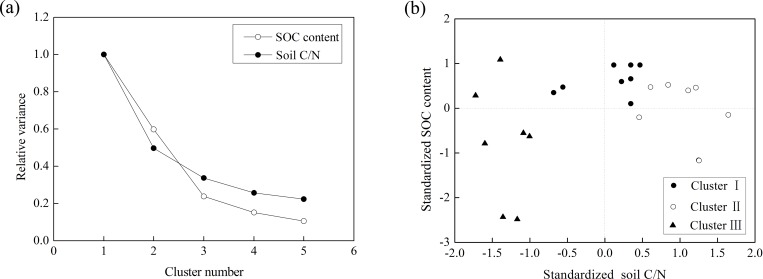
Results of cluster analysis based on soil carbon/nitrogen ratio (soil C/N) and soil organic carbon (SOC) content. (a) Changed pattern of relative variance with the increase in cluster number; (b) Points of the clusters in the standardized soil C/N-standardized SOC space. Here, cluster I, cluster II, and cluster III separated quite clearly from each other.

During cluster analyses, the relative variance of SOC content and soil C/N significantly decreased with an increasing cluster number (Fig 2A). When the cluster number was 3, the relative variance of SOC content and soil C/N decreased to around 25%. The further increase of the cluster number did not cause a significant decline in the relative variance of SOC content and soil C/N. Thus, all the samples were classified into three clusters, according to changes in relative variance and number of available data. Based on the standardized soil C/N and standardized SOC content, Clusters I, II, and III were clearly separated from one another (Fig 2B). Fig 1C shows the spatial distribution of the 23 sample plots in the three clusters. Sample plots belonging to the same cluster indicated a high degree of similarity in their soil properties. Except for the sample plots in Cluster III, spatial continuity was obvious in Clusters I and II (Fig 1C).

### Relationships between biotic factors and vegetation indices

Pearson’s correlation analysis revealed that three biotic factors (i.e., LAI, AGB, and Chl_canopy_) exhibited high correlations for winter wheat and summer maize (Table 1). Therefore, LAI was selected to analyze the relationships between the biotic factors and VIs. Based on regression analysis, EVI consistently demonstrated the optimal linear relationship to LAI, with R^2^ = 0.75 for winter wheat and R^2^ = 0.71 for summer maize (S1 and S2 Figs). However, with increasing LAI, the NDVI of the winter wheat canopy showed obvious saturation, especially when LAI was larger than 3. The CI_red edge_ and EVI greatly improved this problem, and EVI appeared to be the optimal factor among the three VIs. The logarithmic fit quantitatively illustrated this point (S1 Fig). Thus, EVI was selected for the following analysis.

### Relationships between soil respiration and enhanced vegetation index

**Fig 3:**
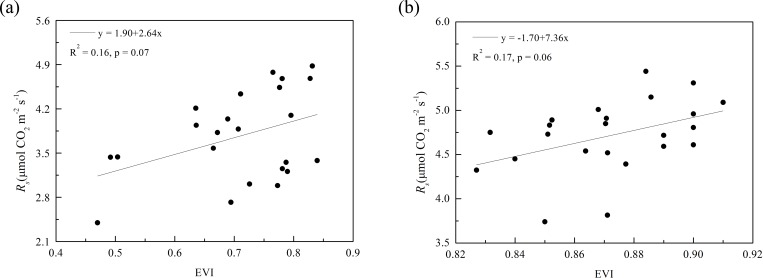
Relationship between soil respiration (*R*_*s*_) and enhanced vegetation index (EVI) for (a) winter wheat and (b) summer maize.

**Fig 4:**
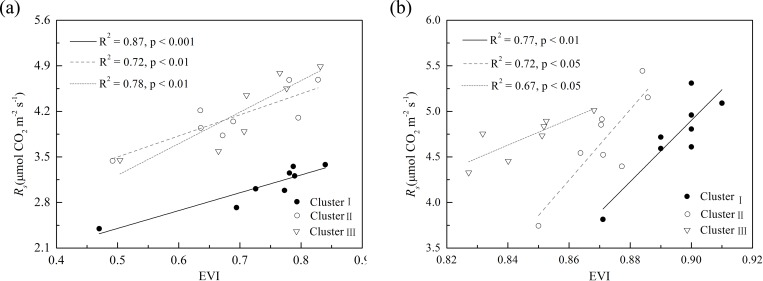
Relationships between soil respiration (*R*_*s*_) and enhanced vegetation index (EVI) based on the data from the three clusters for (a) winter wheat and (b) summer maize. Cluster analysis was conducted based on soil carbon/nitrogen (soil C/N) and soil organic carbon (SOC) content.

After combining all data from the 23 sample plots, EVI did not display a statistically significant relationship with *R*_*s*_ in the sample field under a winter wheat and summer maize rotation (Fig 3). After grouping all the data from the 23 sample plots into three clusters based on the cluster analysis of soil C/N and SOC content, the relationship between *R*_*s*_ and EVI was apparently improved (Fig 4). For the three clusters, the relationship between *R*_*s*_ and EVI could be empirically fitted as a linear function; EVI explained 72%–87% spatial variation of *R*_*s*_ for winter wheat and 67%–77% of that for summer maize (Fig 4). However, the linear fitting functions differed among the three clusters for both crop types; that is, the same increase in EVI corresponded to a significantly different magnitude of variation in *R*_*s*_ in the three clusters.

## Discussion

### Response of soil respiration to soil temperature

The measured *R*_*s*_ (2.40–4.88 μmol CO_2_ m^-2^ s^-1^) was consistent with the range reported from a winter wheat plot (2.58–5.04 μmol CO_2_ m^-2^ s^-1^), close to the present study site [18]. The mean *R*_*s*_ at the jointing period of winter wheat (3.77 μmol CO_2_ m^-2^ s^-1^) in the present study was higher than that in the semi-arid Loess Plateau (approximately 3 μmol CO_2_ m^-2^ s^-1^) [52] and the Tibetan Plateau (1.42 μmol CO_2_ m^-2^ s^-1^) [53] but lower than that in the temperate region of the North China Plain (approximately 5 μmol CO_2_ m^-2^ s^-1^) [54]. At the late jointing stage of summer maize, the measured mean *R*_*s*_ (4.74 μmol CO_2_ m^-2^ s^-1^) was lower than that in the North China Plain (5.47 μmol CO_2_ m^-2^ s^-1^) [18] and higher than that in the Northeast China plain (approximately 3.7 μmol CO_2_ m^-2^ s^-1^) [55]. The present results supported the previous studies, which suggested that *R*_*s*_ of the same crop in different regions might vary with climate and cropland management practices [56], [57].

For the field sampling of winter wheat and summer maize, each *R*_*s*_ measurement was conducted between 09:00 h and 12:00 h (local time) on two continuous sunny days. Within the T_s10_ range of 12.7°C– 14.9°C for winter wheat and 22.1°C– 25.2°C for summer maize, the spatial variation of *R*_*s*_ did not display the predicted increase with the increase in soil temperature (Table 1). This result did not contradict the general positive response of *R*_*s*_ to temperature but indicated that temperature is not necessarily the most important factor in explaining the spatial variation of *R*_*s*_. Previous studies revealed that the spatial variation of *R*_*s*_ within an ecosystem was poorly explained by the spatial variation of soil temperature [25], [27], [43]. In addition, the spatial variation of T_s10_ was low, with CVs of 4.8% and 3.5% for winter wheat and summer maize, respectively. The inadequate temperature range would limit the significant regression of *R*_*s*_ versus temperature [58], [59].

### Response of soil respiration to soil water content

The experimental field in the present study was under strict water management (i.e., irrigating based on crop growth and climate condition) to avoid drought. Approximately 1 week prior to the winter wheat field experiment, the sample field was fully irrigated. The soil water content was considered suitable for winter wheat growth after 1-week soil evaporation and plant water use. In addition, precipitation was prevalent in late July at our study site. After 4–5 days of a heavy rain event at the end of July, we conducted the *R*_*s*_ experiment in the summer maize field. Therefore, the soil water content might be optimal for *R*_*s*_ of winter wheat and summer maize when we conducted field experiments. It was expected that the soil water content did not significantly affect the spatial variation of *R*_*s*_ in the field under a winter wheat and summer maize rotation in the North China Plain. Previous studies also demonstrated that *R*_*s*_ shows minimal response to soil water content for a broad range of near-optimum soil water content [60], [61], [62]. However, the soil water content could become the dominant factor controlling *R*_*s*_ under extremely wet or dry conditions [13], [62], [63].

### Influence of biotic factors on soil respiration

Several factors, such as soil, climate, and human management, affect crop growth in croplands [64], [65]. The soil property is just one of these factors. In the present study, biotic and abiotic factors were evaluated to explain the spatial variation of *R*_*s*_ (Table 1). Biotic factors (i.e., LAI, AGB, and Chl_canopy_) reflected the conditions of crop growth and displayed considerably higher spatial variation (with a mean CV of approximately 23% for winter wheat and 21% for summer maize) than soil properties (with a mean CV of approximately 10%).

Biotic factors affect root respiration and consequently *R*_*s*_ when environmental factors are not limiting for *R*_*s*_ [2], [24], [66]. Typically, the root activity of crops was high at the vegetative growth stage and low at the reproductive growth stage. We conducted field experiments at the late jointing stage of winter wheat and summer maize, which nearly corresponded with the peak of the vegetative growth period. Thus, the proportion of live root respiration accounted for total *R*_*s*_ might be high during our measurement periods. However, we did not obtain the field-measured root respiration to support this assumption. A previous study [67] demonstrated that root respiration accounted for approximately 60% of the total *R*_*s*_ in a winter wheat stand at the late jointing stage; the spatial variability in *R*_*s*_ mainly represented the spatial variability of the autotrophic component. Ding *et al*. [68] demonstrated that autotrophic respiration of a maize cropland could reach up to 70% of the total *R*_*s*_ at the jointing period. Although we did not measured live root biomass for each plot in the present study, we measured live root biomass of winter wheat and summer maize during the growing season at the same study site (in 2011) and a nearby sample plot (in 2010). LAI showed a good linear relationship to live root biomass at the seasonal time scale for winter wheat and summer maize (S3 Fig). Therefore, live root biomass can be inferred from LAI in this study.

### Relationships between spectral vegetation indices and LAI

Among the three VIs, NDVI tends to be saturated at high vegetation densities and showed a poor linear relationship to LAI than EVI and CI_red edge_ for winter wheat and summer maize (S1 and S2 Figs). This trend may be attributed to the fact that EVI and CI_red edge_ improve the canopy background reflectance; both are also more sensitive to variation in dense vegetation than NDVI [69], [70]. As a greenness vegetation index, EVI could serve as a strong proxy for plant productivity [71], [72]. In the present study, the biotic factor LAI was used as surrogate to processes related to total carbon uptake (or plant photosynthesis) by crops. The strong correlation between EVI and LAI supported these assumptions (S1 and S2 Figs).

### Influence of soil properties on the relationship between soil respiration and spectral vegetation index

A single linear function could not describe the relationship between *R*_*s*_ and EVI for winter wheat and summer maize in this study (Fig 3). However, after clustering based on soil C/N and SOC content, the linear relationships between *R*_*s*_ and EVI were significant (p < 0.01) in each cluster for both crops (Fig 4). These findings indicated that the spatial variation of *R*_*s*_ in the present study may be attributed to the spatial variations of soil properties and biotic factors. Similarity, Xu and Qi [28] obtained data from two 20 m × 20 m plots and reported that biological factors and soil properties dominated the spatial variation of soil CO_2_ efflux in a young ponderosa pine plantation. This result also agreed well with Huang et al. [43], who reported that the LAI and SOC content directly affected the spatial variability of *R*_*s*_ during the peak growing season of maize in three counties in North China. However, it should be noted that these results were obtained from a 140 m × 100 m sample area (present study), two 20 m × 20 m plots [28] and three counties [43] because physical and biological controls on soil CO_2_ efflux might differ for ecosystems at large scales. For example, the soil water content could be an important factor affecting the spatial variation of soil CO_2_ efflux at large scales, where the soil drainage class varies across landscapes [16], [73], [74].

In each cluster, the strong linear relationship between EVI and *R*_*s*_ at the late jointing stage of winter wheat and summer maize was mainly caused by background correlation of both quantities with biotic factors [e.g. LAI; S1(C), S2(C) and S4 Figs]. EVI and *R*_*s*_ displayed a strong linear relationship in each cluster (Fig 4) because of the close relationship between plant photosynthesis and *R*_*s*_ [5], [75]. Inconsistencies in the relationships between EVI and *R*_*s*_ before and after the cluster analysis were possibly due to the confounding influences of soil properties. Under the field conditions, the effects of biotic and abiotic factors on *R*_*s*_ are often confounded between each other [55]. The difference between the soil properties in each cluster was reduced by cluster analysis (Fig 2), which clarified the relationship between EVI and *R*_*s*_ (Fig 4). In each cluster, a significant positive linear relationship was observed between EVI and *R*_*s*_ (Fig 4). This observation suggested that *R*_*s*_ was higher at sites with higher photosynthetic capacity when the values of soil properties were maintained at a certain range. This phenomenon agreed with several previous studies, where the spatial variability of vegetation productivity affected spatial variation of *R*_*s*_ in the absence of other restricting factors [20], [24], [76].

The linear relationship between *R*_*s*_ and EVI in each cluster for both winter wheat and summer maize (Fig 4) could not be described by a single function, which indicated that the photosynthetic dependence of *R*_*s*_ was influenced by processes related to soil properties and crop types. With increasing EVI, a different increasing rate of *R*_*s*_ in each cluster was observed (Fig 4) because of the interaction effects of soil (belowground) and vegetation (aboveground) on *R*_*s*_ (S2 Table). For winter wheat and summer maize, significant differences were noted in biotic factors (i.e., LAI, AGB and Chl_canopy_) and soil property factors (i.e., STC content, Soil C/N, and SOC content) between Cluster Ⅰ and Cluster Ⅲ, and between Cluster Ⅱ and Cluster Ⅲ. These results indicated that the application of cluster analysis to minimize differences in soil properties among different clusters may improve the role of remote sensing data as a substitute of plant photosynthesis in semi-empirical *R*_*s*_ models and benefit the acquisition of *R*_*s*_ in cropland ecosystems at large scales.

## Supporting Information

S1 Table Description of 23 sample plots where soil respiration measurements were taken for winter wheat and summer maize. (DOCX)

S2 Table Effect of different clusters on average soil respiration, biotic and abiotic factors for winter wheat and summer maize. Different letters indicate significant differences among clusters (p < 0.05).
(DOCX)

S1 Fig Relationships between leaf area index (LAI) and spectral vegetation indices (VIs) at the jointing stage of winter wheat. The VIs are normalized difference vegetation index (NDVI), enhanced vegetation index (EVI), and red edge chlorophyll index (CI_red edge_). All relationships were statistically significant at p < 0.0001.
(TIF)

S2 Fig Relationships between leaf area index (LAI) and spectral vegetation indices (VIs) at the jointing stage of summer maize. The VIs are normalized difference vegetation index (NDVI), enhanced vegetation index (EVI), and red edge chlorophyll index (CI_red edge_). All relationships were statistically significant at p < 0.0001.
(TIF)

S3 Fig Relationships between leaf area index (LAI) and live root biomass (a) during the 2011 growing season of winter wheat at our study site and (b) during the 2010 growing season of summer maize at a nearby sample plot.
(TIF)

S4 Fig Relationships between soil respiration (*R*_*s*_) and leaf area index (LAI) based on the data from the three clusters for (a) winter wheat and (b) summer maize. Cluster analysis was conducted based on soil carbon/nitrogen (soil C/N) and soil organic carbon (SOC) content.
(TIF)
