# Supplementary material for: Spatial Variation of Soil Respiration in a Cropland under Winter Wheat and Summer Maize Rotation in the North China Plain
Source: PLoS One. 2016 Dec 15;11(12):e0168249. doi: 10.1371/journal.pone.0168249 (PMC5158051; doi:10.1371/journal.pone.0168249)
Supplement: S1 Table — (DOCX) [file pone.0168249.s001.docx]

**S1 Table** Description of 23 sample plots where soil respiration (*R_s_*) measurements were taken for winter wheat and summer maize.

| Plot | Longitude (°) | Latitude (°) | Winter wheat | |  | Summer maize | |
| --- | --- | --- | --- | --- | --- | --- | --- |
|  |  |  | Average value of *R_s_* | SD of *R_s_* |  | Average value of *R_s_* | SD of *R_s_* |
| 1 | 116.4433 | 40.1788 | 3.44 | 0.30 |  | 3.32 | 0.90 |
| 2 | 116.4438 | 40.1779 | 4.54 | 0.31 |  | 4.83 | 1.53 |
| 3 | 116.4436 | 40.1779 | 4.78 | 0.17 |  | 3.88 | 0.60 |
| 4 | 116.4438 | 40.1781 | 4.44 | 0.45 |  | 5.01 | 1.00 |
| 5 | 116.4436 | 40.1781 | 4.88 | 0.58 |  | 4.85 | 1.06 |
| 6 | 116.4433 | 40.1783 | 3.94 | 0.47 |  | 4.91 | 0.92 |
| 7 | 116.4433 | 40.1781 | 4.21 | 0.46 |  | 5.70 | 1.68 |
| 8 | 116.4431 | 40.1781 | 4.68 | 0.25 |  | 4.11 | 0.84 |
| 9 | 116.4431 | 40.1783 | 4.68 | 0.81 |  | 4.39 | 0.99 |
| 10 | 116.4431 | 40.1784 | 4.10 | 0.21 |  | 5.44 | 0.52 |
| 11 | 116.4431 | 40.1786 | 3.83 | 0.40 |  | 4.94 | 1.36 |
| 12 | 116.4431 | 40.1788 | 4.04 | 0.57 |  | 5.12 | 0.71 |
| 13 | 116.4435 | 40.1784 | 2.72 | 0.17 |  | 3.82 | 1.01 |
| 14 | 116.4433 | 40.1786 | 2.99 | 0.25 |  | 5.00 | 0.87 |
| 15 | 116.4435 | 40.1786 | 3.01 | 0.26 |  | 4.72 | 0.69 |
| 16 | 116.4433 | 40.1779 | 3.21 | 0.26 |  | 5.53 | 0.92 |
| 17 | 116.4435 | 40.1783 | 3.38 | 0.15 |  | 4.82 | 0.43 |
| 18 | 116.4433 | 40.1784 | 2.40 | 0.21 |  | 4.78 | 0.87 |
| 19 | 116.4437 | 40.1783 | 3.35 | 0.36 |  | 4.59 | 1.29 |
| 20 | 116.4438 | 40.1784 | 3.25 | 0.42 |  | 4.81 | 0.83 |
| 21 | 116.4438 | 40.1786 | 3.58 | 0.58 |  | 4.73 | 1.13 |
| 22 | 116.4438 | 40.1787 | 3.44 | 0.30 |  | 5.45 | 0.73 |
| 23 | 116.4435 | 40.1788 | 3.88 | 0.35 |  | 4.18 | 0.97 |
