# Supplementary material for: Spatial Variation of Soil Respiration in a Cropland under Winter Wheat and Summer Maize Rotation in the North China Plain
Source: PLoS One. 2016 Dec 15;11(12):e0168249. doi: 10.1371/journal.pone.0168249 (PMC5158051; doi:10.1371/journal.pone.0168249)
Supplement: S2 Table — Different letters indicate significant differences among clusters (p < 0.05). (DOCX) [file pone.0168249.s002.docx]

**S2** **Table** Effects of different clusters on the soil respiration (*R_s_*), as well as the biotic and abiotic factors, for winter wheat and summer maize. Different letters (i.e., a, b, and c) indicate significant differences in the *R_s_* or biotic and abiotic factors among three clusters (p < 0.05).

|  | Winter wheat | | |  | Summer maize | | |
| --- | --- | --- | --- | --- | --- | --- | --- |
|  | Cluster Ⅰ | Cluster Ⅱ | Cluster Ⅲ |  | Cluster Ⅰ | Cluster Ⅱ | Cluster Ⅲ |
| *R_s_* | 3.04 a | 4.11 b | 4.22 b |  | 4.76 a | 4.74 a | 4.70 a |
| ***Biotic factors*** | | | | | | | |
| LAI | 3.33 a | 3.11 a | 2.61 b |  | 3.01 a | 2.86 a | 2.64 a |
| AGB | 0.43 a | 0.43 a | 0.40 b |  | 0.28 a | 0.27 a | 0.24 b |
| Chl_canopy_ | 1.45 a | 1.35 a | 1.14 b |  | 1.26 a | 1.17 a | 1.05 b |
| ***Environmental factors*** | | | | | | | |
| SWC_20_ | 25.0 a | 26.6 a | 28.5 a |  | 35.2 a | 37.1 a | 38.2 a |
| T_s10_ | 13.2 a | 14.1 a | 13.5 a |  | 23.45 a | 23.31 a | 23.63 a |
| ***Soil property factors*** | | | | | | | |
| STN content | 0.17 a | 0.16 a | 0.16 a |  |  |  |  |
| STC content | 1.18 a | 1.16 a | 1.02 b |  |  |  |  |
| Soil C/N | 6.81a | 7.10a | 6.35b |  |  |  |  |
| SOC content | 1.18a | 1.08a | 0.95b |  |  |  |  |

*R_s_* is the soil respiration (μmol CO_2_ m^-2^ s^-1^), LAI is the leaf area index, AGB is the aboveground biomass (kgm^-2^), Chl_canopy_ is the canopy chlorophyll content (g m^-2^), SWC_20_ is the soil water content at 0–20 cm depth (%), T_s10_ is the soil temperature at 10 cm depth (°C), STN content is the soil total nitrogen content (%), STC content is the soil total carbon content (%), soil C/N is the soil carbon/nitrogen ratio, and SOC content is the soil organic carbon content (%).
