# Supplementary figures and images for: Spatial Variation of Soil Respiration in a Cropland under Winter Wheat and Summer Maize Rotation in the North China Plain
Source: PLoS One. 2016 Dec 15;11(12):e0168249. doi: 10.1371/journal.pone.0168249 (PMC5158051; doi:10.1371/journal.pone.0168249)

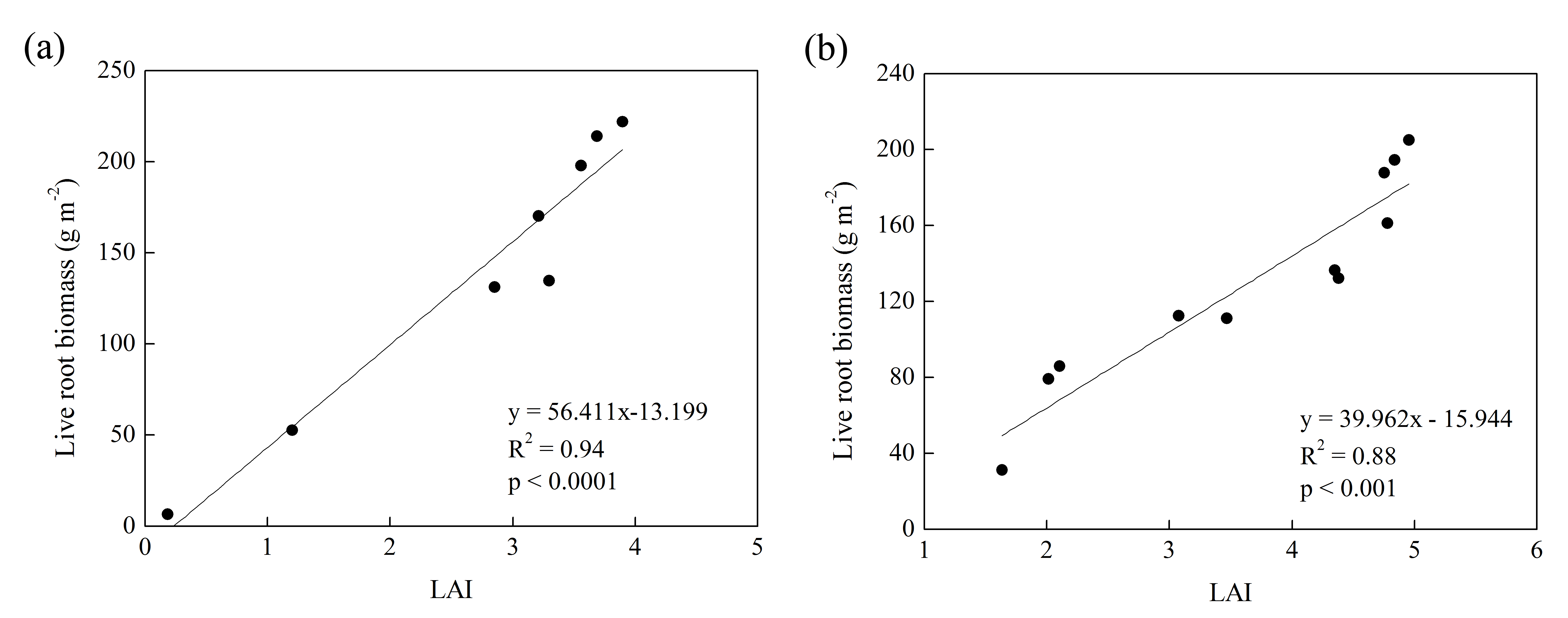

Supplement: S3 Fig — Relationships between leaf area index (LAI) and live root biomass (a) during the 2011 growing season of winter wheat at our study site and (b) during the 2010 growing season of summer maize at a nearby sample plot. (TIF) [file pone.0168249.s005.tif]
